# Cold Feet

**Published:** 1887-10-22

**Authors:** 


### The Doctor's Pulpit.

### COLD FEET.

In human affairs a very small thing often makes the difference between happiness and the want of it. Such a trouble as cold feet may turn all the grapes of life into green gooseberries. Samson and Ajax and Captain Webb probably understood as little about cold feet as they did about embroidery or any other occupation purely feminine. The people of strong build and high-pressure circulation have small sympathy with their fellow-mortals of slighter structure and slower arterial motion ; still less can they enter into the feelings of many women, who from inherited peculiarity or acquired habit never feel really warm. Coldness of the lower extremities cannot be called a disease ; but it is a peculiarity which causes serious discomfort, and may give rise to disease. In some people it is so pronounced as to make sleep difficult or even impossible ; and in others it results in painful and disabling chilblains. These things appear small, no doubt, and hardly worth writing about ; but that is a mistake. Whatever interferes with the feeling of perfect well-being, or incapacitates man, woman, or child for duty and pleasure, is well worthy of consideration, and, if possible, of removal.

A little thought will enable us to go to the root of the matter, and so to thoroughly understand the subject in all its bearings. It is much better to do that than to offer some "hey presto" specific which promises to cure everybody in three or four applications. Specifics as a rule mean knavery, and if people would only take the trouble to think out for themselves the nature and cause of their own ailments, the knaves would have to pursue another calling, to the very great advantage of their former dupes. What are the causes of cold feet? The most frequent cause probably is inherited tendency, and the next, perhaps, laziness. A man, or still oftener a woman, is a chilly subject because one of her parents—generally her mother —was chilly before her. Why should women suffer more from this kind of feebleness than men ? For various reasons : one being that they are of a less robust muscular organisation, and so the blood-pumping is less energetically carried on, and its force fails sooner. Another reason is their indoor life, which prevents their blood from being highly oxidised, and also depresses the nervous system, thus still further enfeebling the "pumping" economy. There are also other reasons which will readily occur to the thoughtful. The general proposition is indisputable, that women more than men suffer from cold extremities, and from a variety of troubles which are usually associated therewith.

So far everybody is agreed : we have now to ask what is to be done by way of cure ? Let us revert for a moment to our list of "causes." These were said to be principally two—"inherited tendency" and "laziness." With regard to the former, many people will be ready to give up the contest, because they will think that if the tendency is "inherited" it is natural to them, and "people cannot change their nature." Why cannot people "change their nature" ? As a matter of fact people can, and constantly do. The whole of life, when lived on the higher plane, is a constant effort to intelligently change the nature ; and it is often quite as easy, perhaps easier, to change the physical than to change the moral nature. Nothing is more common than to see men and women who in youth were brisk, vigorous, and bustling country children, sink down, after years of indoor town life, into timid, querulous, and feeble valetudinarians, who hardly dare to walk alone in the streets for fear of being run over and killed. On the other hand, it is sometimes, though less frequently seen, that a man of strong will converts his originally feeble and ineffective physical organism into a tough, enduring, and most capable working instrument. John Wesley was a conspicuous example of the latter class. He was small and feeble, with a tendency to consumption, and frequently during his lifetime spat blood ; yet, by regularity of life and work, and constantly living in the open air, he managed to reach a ripe old age, and to accomplish as much work for his generation as dozens of ordinary men.

The inherited tendency to cold feet, and all that is included in that peculiarity, is to be overcome by reason, and by the reason of the owner of the feet. Nothing else can do it. Sense and will : these are the rare drugs which will effect a cure. But where is the pharmacy that dispenses such golden treasures of physic? That is where the difficulty lies. If a woman—for the sufferer is mostly a woman—have intelligence and determination enough to use regularly and for a sufficient length of time the methods now to be suggested, she will, unless too old or feeble, almost certainly effect a cure. These methods are friction, exercise, hygiene, and steel. The vigorous rubbing of the cold extremities with the hand anointed with plain oil for half-an-hour morning and evening ; a brisk walk for half-an-hour after breakfast, and four more such walks for a quarter of an hour at regular intervals during the day ; wise hygienic arrangements which include suitable clothing for the body, and also for the legs and feet, both day and night ; and the periodical taking of large doses of some suitable preparation of steel—these will infallibly cure, if cure be possible. The very same methods are applicable when the affection is the result of laziness.

"Thank you for nothing," says many a reader. "We knew all that before ; and who is going to be at the trouble of carrying out such a course of treatment as that ?" Most true ! As we before said : that is exactly where the whole difficulty lies. The doctor's pulpit has precisely the same opponents to fight against as every other pulpit. People want to be both virtuous and self-indulgent : but they cannot. And they also want to be both healthy and self-indulgent : but they cannot. Reason, knowledge, and obedience to their dictates are the methods of virtue ; they are also the methods of health. The coincidence is worth a moment's consideration. It suggests that there is a fixed order in the world, and that the order is intelligent : it suggests the ideas of reward and punishment, and that an impartial justice presides over the distribution : it suggests law—stern and unyielding, which no power or persuasion of man, be he great or small, a Crœsus or a Lazarus, can for one moment bend to his wishes. A wise man will duly take all these things into account: a fool will go his own way —and reap the consequences.

A few people are too old and feeble to be cured; they must be comforted. Warm flannel clothing, and boots lined with flannel; regular and nourishing diet; frequent courses of steel medicine; as much fresh air as possible, and on foot when it is at all practicable; hot bottles for the carriage, and a hot bottle for the bed,—these and similar comforts are to be encouraged. Some people think hot bottles do harm by "tendering" the feet, and so on. It is to be borne in mind that whatever harm comforts may do in this connection, the cold feet produced by the want of them will do infinitely more harm still.

